# Population Pharmacokinetic Modelling of Orally Administered Doxycycline to Rabbits at Different Ages

**DOI:** 10.3390/antibiotics10030310

**Published:** 2021-03-17

**Authors:** Rositsa Mileva, Anton Rusenov, Aneliya Milanova

**Affiliations:** 1 Department of Pharmacology, Animal Physiology and Physiological Chemistry, Faculty of Veterinary Medicine, Trakia University, 6000 Stara Zagora, Bulgaria; rositsa.mileva@trakia-uni.bg; 2 Department of Internal Noninfectious Diseases, Faculty of Veterinary Medicine, Trakia University, 6000 Stara Zagora, Bulgaria; vetroussenov@abv.bg

**Keywords:** doxycycline hyclate, population pharmacokinetics, rabbits

## Abstract

Doxycycline is a well-tolerated tetracycline antibiotic, registered for use in rabbits and administered for treatment of bacterial infections in this animal species. Nevertheless, the available pharmacokinetic data are limited and this study aimed to investigate the pharmacokinetics of orally administered doxycycline in mature and immature rabbits by application of the population approach. The rabbits were treated orally with doxycycline hyclate (5 mg/kg bw) in the form of a solid gelatin capsules. Free plasma concentrations were determined with HPLC analysis with Photodiode array detection. The estimated typical value of volume of distribution (tvV), total body clearance, and absorption rate constant were 4.429 L/kg, 1.473 L/kg/h, and 0.257 h^−1^, respectively. The highest between-subject variability (BSV) of 69.30% was observed for tvV. Co-variates such as body weight, age, and biochemical parameters did not improve the tested model and did not contribute to explanation of the BSV. The population pharmacokinetic model of the orally administered doxycycline in rabbits should be further developed by addition of data from more animals treated with higher doses. An oral dose of 5 mg/kg could ensure percentage of the time from the dosing interval during which the concentration is above minimum inhibitory concentration (MIC) %fT > MIC of 35% if MIC of 0.18 μg·mL^−1^ and a dosing interval of 12 h is assumed which does not cover criteria for rational use of antibiotics.

## 1. Introduction

The importance of rabbits in veterinary medicine is increasing due to the high nutritional value of rabbit meat and their breeding as pets [1,2]. This animal species often suffers from infections caused by pathogenic bacteria such as *Pasteurella multocida, Staphylococcus aureus*, *Bordetella bronchiseptica, Salmonella* spp., *Escherichia coli, Clostridium* spp., and *Pseudomonas* spp. [3,4]. These pathogens are sensitive to antibiotics often used in veterinary practice such as broad spectrum penicillins, macrolides, lyncosamides, amynoglycosides, fluoroquinolones, sulfonamides, and tetracyclines [5,6,7,8,9,10]. Administration of antimicrobials is not well tolerated by rabbits and treatment with penicillins, cephalosporins, macrolides, and lincosamides is associated with enteritis [11] as they destroy normal gastrointestinal microbiota allowing overpopulation of pathogenic bacteria such as *E.coli* and *Clostridium spiroforme* [12].

Tetracycline antibiotics, especially from newer generations, can be applied in rabbits without fatal disruption of the balance of gastrointestinal microbiota [13]. Doxycycline is a tetracycline antibiotic with high activity against the pathogens causing infections in this animal species. The maximum residue limit (MRL) values for different tissues are extrapolated from other animal species [14] and doxycycline is registered for use in rabbits in some EU countries [15]. Increasing problems with emergence of bacteria resistant to antibiotics, including tetracyclines, require taking into account that domestic rabbits may be carriers of phenotypically antimicrobial-resistant bacteria as a part of their microbiome, and genes encoding antimicrobial resistance (AMR) [16]. Considering the mentioned risks, a precise administration of doxycycline in rabbits, based on knowledge about pharmacokinetics and pharmacodynamics, is needed. The available data about pharmacokinetics of doxycycline in this animal species are limited to two publications. As in other animal species, the elimination half-life (t_1/2_) of intravenously administered doxycycline to New Zealand rabbits at a dose rate of 5 mg/kg was long and the values of total body clearance (Cl) were relatively low at 8.83 ± 2.10 h and 0.46 ± 0.10 L/h/kg, respectively [17]. Administration of various extravascular dosage forms showed that doxycycline was absorbed with absolute bioavailability of 49.13 ± 14.69% − 51.43 ± 4.50% for suppositories and relative bioavailability of chitosan microcapsulated suspension of 289.4% when compared to water solution of the antibiotic [17,18]. Different values of C_max_ between 1.10 ± 0.00 μg·mL^−1^ and 2.06 ± 2.96 μg·mL^−1^ were reported for the drug in studies conducted with various dosage forms and doses. These concentrations were achieved at T_max_ between 0.55 ± 0.33 and 0.70 ± 0.48 h for suppositories, 3.00 h for the solution and 12.00 h for chitosan microcapsulated suspension [17,18]. Despite the limited data in the available literature about the pharmacokinetics of doxycycline in rabbits, this antibiotic is often prescribed at dose rates from 2.5 to 6 mg/kg [4,14]. The cited investigations were carried out with mature rabbits. No data are published about the pharmacokinetics of doxycycline in young animals, which often suffer from infections caused by the above-mentioned pathogens.

A problem encountered during pharmacokinetic experiments is related to ethical reasons and the difficulties of obtaining a sufficient number of samples due to the small size of rabbits grown for classical pharmacokinetic studies. The population pharmacokinetic analysis can be successfully applied in cases when only few samples can be obtained from an individual animal [19]. Therefore, the aim of the current study was to characterize the pharmacokinetics of doxycycline after oral administration in rabbits by using the population approach.

## 2. Results

**Figure 1:**
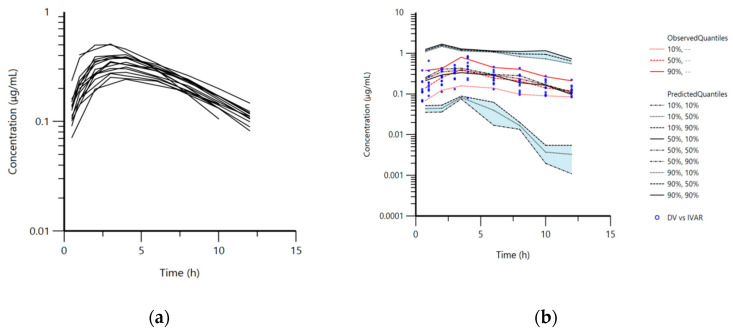
(**a**) Semi-logarithmic spaghetti plot of disposition curves of orally administered doxycycline hyclate in rabbits (*n* = 16) at a dose rate of 5 mg/kg and (**b**) visual predictive check (VPC) plot depicting the observed quantiles (10, 50, and 90%) and corresponding predictive quantiles. Light pink and light blue areas correspond to the 95% confidence interval of the three predicted quantiles.

The animals tolerated well the oral application of doxycycline in the form of capsules. The maximum plasma concentrations (C_max_) and the time of C_max_ (T_max_) in mature rabbits read from the observed data, were 0.58 ± 0.15 μg·mL^−1^ and 3.40 ± 0.96 h, respectively. These values for immature animals were 0.60 ± 0.38 μg·mL^−1^ and 3.63 ± 1.33 h. Plasma concentrations were below the limit of quantification (LOQ) 12 h after the treatment in mature rabbits (Figure 1a). Doxycycline was not found in plasma samples in rabbits from both groups 24 h after treatment.

**Figure 2:**
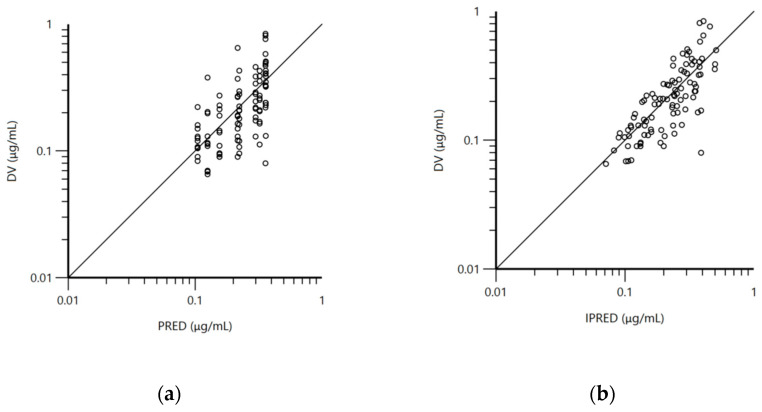
(**a**) Logarithmic plot of the dependent variable (DV, doxycycline concentrations) vs. population predicted doxycycline concentrations (PRED) and (**b**) logarithmic plot of the dependent variable (DV, doxycycline concentrations) vs. individual predicted doxycycline concentrations (IPRED).

The pharmacokinetic model used for the analysis was a one-compartment model with first-order absorption and elimination. The structural model was adequate to describe doxycycline disposition in mature and immature rabbits in the current experiment and is shown on the visual predictive check plot (Figure 1b). The data for age, body weight, and biochemical parameters were not included as co-variates in the final model because of lack of significant improvement of the model. The mean plasma concentrations of doxycycline for both groups of rabbits (Table S1), blood biochemical parameters (Table S2), total protein, albumin, alanine transaminase (ALT), aspartate transaminase (AST), and lactate dehydrogenase (LDH) are presented in the Supplementary File 1. Visual inspection of the goodness-of-fit plot of the population predicted concentration (Figure 2a) and plot of the individual predicted concentrations (Figure 2b) versus the observed doxycycline concentrations demonstrated no major bias in the population model. The evenly distributed data about the line of identity on Figure 2a,b confirmed this observation.

Typical values (population mean, θ) of the primary structural parameters of the population model, the secondary pharmacokinetic parameters, their standard errors, and the SD of the residuals are shown in Table 1.

The random effects, the between-subject variability (BSV) and shrinkage are presented in Table 2. The values of shrinkage indicated that the data set was large enough and that the model could be further developed by addition of data from more animals. The BSV values for-ka and Cl suggested homogenous exposure to doxycycline. The volume of distribution and clearance in this model were actually apparent volume of distribution per fraction absorbed (V/F) and Cl/F for an oral dose. These primary parameters were affected by extent of absorption. In contrast to the BSV value for Cl and ka, the value of V was higher—69.30%.

## 3. Discussion

Rabbits belong to the group of “minor species”, animal species which have economic significance and for which there are not enough antibacterial drugs approved for treatment of bacterial infections. Emergence of resistant bacteria and increasing problems with the efficacy of the available antibiotics require knowledge of pharmacokinetics with consideration of inter-individual variability as a first step of establishment of pharmacokinetic/pharmacodynamic (PK/PD) cutoff value [20]. Thus, the current study established a population pharmacokinetic model and calculated typical pharmacokinetic parameters with information about between-subject variability.

The applied HPLC method with Photodiode array (PDA) detection showed similar sensitivity to the methods used in the published pharmacokinetic studies in rabbits [17,18]. The method of extraction used in the current study allowed determination of free doxycycline concentrations. A previously conducted study in our lab revealed that an additional purification step, performed by ultra-centrifugation through filters with pore size of 0.45 µm, required further correction of the concentrations of 0.5–1 µg·mL^−1^ and 0.25 µg·mL^−1^ by 5–6% and 24.6%, respectively [21]. Therefore the measured concentrations were not further corrected.

Doxycycline was absorbed after oral administration in immature and mature rabbits and measurable concentrations were found 0.5 h after the treatment. The C_max_ values were obtained four hours after the doxycycline administration in both groups of rabbits. The relatively low value of ka, calculated with the current model, confirmed a slow absorption rate after oral administration of doxycycline in rabbits, administered as capsules. The pharmacokinetic studies of doxycycline in rabbits are scarce which makes the comparison of the data from the current investigation with other studies difficult. The available literature concerns pharmacokinetics of doxycycline hyclate in the form of a solution, a chitosan microcapsulated suspension, and suppositories [17,18]. The absorption rate of the microcapsulated suspension of 0.2 ± 0.11 h^−1^ was similar to tvka of 0.257 h^−1^. Faster absorption, with value of ka 1.26 ± 0.71 h^−1^, was observed after oral administration of a solution of doxycycline hyclate in rabbits at a dose rate of 20 mg·kg bw [18]. In comparison to our data, an almost twice-higher value of C_max_ of 1.54–2.06 µg·mL^−1^ achieved at T_max_ of 0.55–0.7 h was reported after administration of the antibiotic as suppositories at a dose of 10 mg/kg bw [17]. The faster absorption of doxycycline at higher concentrations in the form of suppositories can be explained by the advantages of the rectal route of administration and by the higher dose [22]. Wistar rats, as an animal species close to rabbits, showed ka value of 0.26 ± 0.03 h^−1^ and higher C_max_ value of 3.20 ± 0.65 µg·mL^−1^ after treatment with a solution of doxycycline hyclate at an oral dose of 10 mg/kg [23].

The primary population pharmacokinetic parameter tvV was in agreement with the results for V/F of 3.11–3.82 L·kg^−1^ obtained for other drug dosage forms orally administered in rabbits and with classical pharmacokinetic modeling [18]. The other primary pharmacokinetic parameter tvCl was similar to Cl/F of 1.88 ± 0.08 L/kg/h reported by Fu et al. [18] after oral administration of the antibiotic as a solution at a dose of 20 mg/kg bw. In contrast to these values, Cl/F was 0.44–0.62 L/kg/h when doxycycline was used as suppositories or as a suspension, respectively [17,18]. It is difficult to compare the value of the secondary pharmacokinetic parameter k_10_ due to administration of different doses, drug formulations, and treatment routes in the published studies with rabbits. The published values of t_1/2el_ of doxycycline hyclate in rabbits were between 2.12 h and 9 h, depending on the listed factors [17,18]. The elimination of doxycycline in mature and immature rabbits was fast and indicated that a single oral dose of 5 mg/kg was not high enough to guarantee keeping the concentration higher than 0.25 µg·mL^−1^ during the whole dosing interval. Despite the specific behavior of rabbits, coprophagy [24], and excretion of doxycycline predominantly through the feces, the levels of doxycycline were below the limit of detection (LOD) in both groups 24 h after the treatment. The range of the applied doses of doxycycline hyclate (from 5 to 20 mg/kg) and various drug formulations does not allow for reliable comparison of the values of AUC. Single intravenous administration of doxycycline to New Zealand rabbits at a dose rate of 5 mg/kg resulted in AUC_0-∞_ of 11.47 ± 2.91 µg/mL·h [17]. Similar results for AUC_0-∞_ from 11.27 ± 3.37 to 11.79 ± 1.03 µg/mL·h were obtained with administration of suppositories, containing 10 mg/kg doxycycline and prepared with polyethylene glycol or cocoa butter, respectively [17]. Oral administration of the antibiotic as a solution at a dose rate of 20 mg/kg resulted in AUC_0–∞_ of 11.83 ± 0.19 µg/mL·h [18]. The value of AUC (3.40 µg/mL·h) in our study was lower than the reported data which may be attributed to the applied lower dose of 5 mg/kg and probably, to the different degree of absorption of the antibiotic.

The tested model shows that high inter-individual variations can be expected in the volume of distribution. The model could not be improved although it tested the effect of several co-variables, hence they could not be used for explanation of the variability in doxycycline disposition between individual rabbits. The population analysis indicated that a larger number of rabbits should be included in further development of the model in order to obtain a more complete data set and to attempt to explain between-subject variability. This step would help to estimate more precisely the value of individual eta, even with a limited number of samples from an animal [19,25], as well as to discover the main source of variability. The identification of between-subject variability can be useful in designing proper dosing regimens and in optimizing antimicrobial treatment.

The results of our study, in which an oral dose of 5 mg/kg was administered to immature and mature rabbits, revealed that the often-recommended oral dose [4,14] (pp. 137–177) may not be high enough to guarantee achievement, in all rabbits, of effective concentrations against less susceptible bacterial species with MIC > 0.25 µg·mL^−1^. Data on the susceptibility of pathogenic bacteria, that is relevant for rabbits, are scarce, therefore %fT > MIC was calculated by assuming a probable MIC value of 0.18 µg·mL^−1^ for *Pasteurella spp*. [26]. Despite imperfections in the method of assuming a probable value of MIC, the estimated percentage of the dosing interval time during which free plasma concentrations were higher than MIC, %*f*T > MIC, was 35% from a τ of 12 h. Based on the power of population modelling, the current pharmacokinetic model could be further developed by adding new data for rabbits treated with a higher dose of doxycycline. Apart from improvement of the population pharmacokinetic model, further studies should supply information about real MIC values for pathogens relevant for rabbits, identify target PK/PD values, and establish a PK/PD cut off value, based on the steps outlined by EuVetCAST procedure [27].

## 4. Materials and Methods

### 4.1. Drugs and Reagents

A commercial formulation of doxycycline hyclate, HydroDoxx 500 mg/g oral powder (Huvepharma, Bulgaria) was used for treatment of the animals. The drug was administered at the dose rate of 5 mg·kg^−1^ bw. The powder was weighed in individual oral gelatin capsules, containing the exact dose according to the individual body weight of the rabbits. The capsules were filled on the day before treatment.

All reagents and solvents were HPLC grade and were provided by Sigma-Aldrich (St. Louis, MO, USA): acetonitrile CHROMASOLV^®^, HPLC grade, ≥99.9% purity, methanol HiPerSolv CHROMANORM for HPLC isocratic grade, oxalic acid 98% purity (Sigma Chemical Co., St. Louis, MO, USA), ethylenediaminetetraacetic acid disodium salt dihydrate 99.0–101.0% (Na_2_H_2_EDTA × 2H_2_O) and trifluoroacetic acid ReagentPlus^®^, 99% purity. Analytical standards doxycycline hyclate with purity ≥ 98% and oxytetracycline hydrochloride ≥ 95% crystalline were used for preparation of standard curves during HPLC analysis.

### 4.2. Animals

Crossbred New Zealand x Californian broiler rabbits (*n* = 18) were enrolled in the experiments. The animals were allocated in two groups according to the age. One of the groups included six mature 5-month-old rabbits with mean body weight 3.55 ± 0.30 kg. The other group consisted of 12 immature 70-day-old rabbits with mean body weight 2.03 ± 0.21 kg. The animals were obtained from the Production experimental base to the Agricultural Institute-Stara Zagora, Bulgaria. The animals were housed in the biobase unit at the Faculty of Veterinary Medicine, Trakia University. Mature rabbits were placed in individual cages and the immature rabbits were divided in groups of four and allocated to three cages, according to the species requirements. Granulated feed (Melchran EOOD, Stara Zagora, Bulgaria) and water were supplied ad libitum. The animals were clinically healthy. The experiments started after a 10-day acclimatization period. After the end of the experiment, the animals were reared at the biobase unit.

### 4.3. Experimental Design

The animals were weighed one day before the treatment. Blood samples were taken with vacutainers (2.5 mL lithium heparin, FL Medical, Torreglia, Italy) for determination of biochemical parameters total protein, albumin, alanine transaminase (ALT), aspartate transaminase (AST) and lactate dehydrogenase (LDH) on the same day. Biochemical analysis was performed on the Biochemistry analyzer BS-120 Mindray (P.R. China) at the Clinical Laboratory, Laboratory and Diagnostic Center, Trakia University.

Doxycycline hyclate was administered orally at a dose rate of 5 mg/kg bw in the form of hard gelatin capsules. The rabbits were treated with a single dose with the help of 2 mL syringes with cut apex between 7:00 and 8:00 a.m. Two rabbits did not swallow the capsule and expelled it with damaged integrity and shell. They were excluded from the experiment because we were not sure how much of the drug had reached gastrointestinal tract. The animals had free access to feed and water at the day of the experiment.

Blood samples from the mature rabbits were obtained from v. auricularis before the treatment and at the following intervals after the treatment: 0.5, 1, 2, 3, 4, 6, 8, 10, 12, and 24 h. The immature rabbits were divided in two groups of five animals each. Blood samples from group 1 (1st to 5th immature rabbits) were taken at 0.5, 2, 4, 8, and 12 h after treatment. Blood samples from the second group (6th to 10th immature rabbits) were obtained at 1, 3, 6, 10, and 24 h after treatment. The samples were immediately placed in Eppendorf tubes containing heparin-sodium and were centrifuged at 1500× *g* for 10 min. Blood samples from untreated rabbits were obtained for preparation of standard solutions with vacutainers (2.5 mL lithium heparin, FL Medical, Italy). Plasma was transferred to a new tube and stored at −25 °C until analysis.

### 4.4. HPLC Analysis

HPLC analysis with PDA detection was used for determination of doxycycline concentrations in plasma. The applied method is described in detail by Mileva [21]. Briefly, 15 µL internal standard (IS, oxytetracycline hydrochloride 11 μg/mL) and 19.5 µL trifluoroacetic acid (TFA) to precipitate proteins was added to an aliquot of 150 µL plasma sample. Samples were vortexed for 1 min and centrifuged at 10,800× *g* at 22 °C for 10 min. The clear upper layer was placed in HPLC vials and 20 µL was injected into the HPLC system (Thermo Fisher Scientific Inc., Waltham, MA, USA). The drug separation was achieved by Hypersil Gold column (5 μM, 150 × 4.6 mm). The analytical equipment consisted of a PDA detector (Surveyor, Thermo Fisher Scientific Inc., USA) set at a wavelength of 345 nm, a Surveyor LC Pump Plus, and a Surveyor Autosampler Plus. The mobile phase consisted of acetonitrile, methanol, 0.02 M oxalic acid, and 0.02 M Na_2_H_2_EDTA × 2H_2_O (20:15:64:1, *v*/*v*/*v*/*v*). The flow rate was 1.0 mL.min^−1^. The retention times were 2.7 min for oxytetracycline and 5.66 min for doxycycline. ChromQuest Chromatography Data System (Thermo Fisher Scientific Inc., USA) was used for peak area integrations.

The method was validated in compliance with the requirements of the international guidelines [28,29,30]. The calibration curves were built using plasma samples from untreated rabbits spiked at six concentrations of doxycycline (0.125, 0.25, 0.5, 1, 2.5, and 5 µg·mL^−1^). IS was added during preparation of the samples for calibration curves. The standard curve was linear within the range of the used concentrations (R^2^ = 0.9981) and goodness-of-fit was proved by the lack of fit test (*p* > 0.08). The values of limits of detection (LOD) and quantification (LOQ) were calculated according to equations described by Shabir [28] and were 0.05 and 0.15 µg·mL^−1^, respectively. The accuracy was between 90.55 and 105.22% for the concentration from 1 µg·mL^−1^ to 0.25 µg·mL^−1^, respectively. The mean intra- and inter-day precision (RSD%) values were 4.88 and 10.48. The extraction recovery of doxycycline was >83.77%.

An additional purification step of plasma samples spiked with three doxycycline concentrations was used to determine the percentage of plasma protein binding after the protein precipitation step. The extraction of the antibiotic was performed according to the procedure described above. Plasma samples with low (0.25 μg·mL^−1^), medium (0.5 μg·mL^−1^), and high (1.0 μg·mL^−1^) concentrations of the antibiotic were subjected to ultrafiltration by using Ultrafree^®^−MC Centrifugal Filters with polytetrafluoroethylene (PTFE) membrane with 0.45 μm pore size (Merk, Millipore). Subsequently, the samples were centrifuged for 4 min at 10,800× *g* at 22 °C and 20 μL ultra-filtrate was injected into the HPLC system. The binding percentage after this purification step was calculated according to the manufacturer’s instructions and according to the following equation:

% = [(conc NF − conc F)/conc NF] × 100(1)

where conc NF was the measured concentration in the sample before the filtration and conc F was the measured concentration after ultra-filtration.

### 4.5. Population Pharmacokinetic Analysis

Population pharmacokinetic analysis was carried out by using Phoenix NLME version 8.3 (Certara, St. Louis, MO, USA). Population pharmacokinetic parameters were computed by application of nonlinear mixed effects (NLME) approach. The one-compartment model was chosen after visual inspection of plots for all rabbits on the basis of the Log Likelyhood (-2LL) and the Akaike information criterion (AIC) to identify the model that fitted best to the observed data. Additionally, we tried to identify the sources of inter-individual variability and co-variates were incorporated in the model. The effects of different co-variates and their combinations on pharmacokinetic parameters were tested. Finally, a model without covariates was chosen based on the results of -2LL and likelihood ratio test (LRT), and the AIC. The model comparer tool of the software was also used. The LRT test was applied for statistical evaluation of goodness-of-fit of the models in order to compare more complex models to the basic model and the critical value of the χ2 distribution considered for a given nominal risk of 0.05. Finally the one-compartmental model without covariates was selected with primary estimated parameters absorption rate constant (ka), volume of distribution (V), and clearance (Cl). The terminal slope k_10_, elimination half-life, and area under the curve were computed with classical equations.

The elimination rate constant k_10_ was calculated according to the Equation (2):

k_10_ = tvCl/tvV (2)

where tvCl is population value for Cl and tvV is population value for V.

The elimination half-life was computed according to the Equation (3):

t_1/2el_ = log(2)/k_10_ (3)

The area under the curve was computed according to the Equation (4):

AUC = Dose/tvCl(4)

where Dose is the applied dose and tvCl is population value for Cl.

The between-subject variability (BSV) was modeled using an exponential model and the pharmacokinetic parameters (V, Cl, and ka) for every subject (*i*th animal) were defined as follows:

V*i* = tvV × Exp(η*_i_*) (5)

where V*i* is the volume of distribution for the *i*th animal, tvV (referred also as θ) is the population V, and η*i* (eta) is the deviation associated with the individual animal *i*^th^ from the computed population value of tvV. The other two parameters were calculated with the same algorithm. Normal distribution of etas with a mean of 0 and a variance (ω^2^) was assumed. Consequently, individual parameters and their etas could be correlated. Equation (6) was used for calculation of coefficient of variation (CV%):

(6) $$\mathrm{CV}\;\mathrm{volume}\;(\%)\;=\;100\times \sqrt{\exp\left(\omega^{2}volume\right)-1}$$

The shrinkage of random effects towards the means was defined according to the following equation:

(7) $$\mathrm{Shrinkage}\;=\;1-\mathrm{SD}(\eta j)/\sqrt{\omega_{j,j}}$$

where SD(η*j*) is the empirical standard deviation of the *j*th (observation) η over all Nsub subjects, and *ωj,j* is the estimate of the population variance of the *j*th random effect, *j* = 1, 2, …, Neta.

Multiplicative (proportional) residual error was used:

C*t* = *f* (θ, Time) × (1 + ε) (8)

where ε is with a mean of zero, and a variance σ^2^.

The first order conditional estimation extended least squares (FOCE-ELS) methodology was used for analyses. It was based on minimizing an extended least squares objective function representing the FOCE approximation to the negative log of the marginal likelihood as a function of (θ, σ-standard deviation, ω). Censored data were not included in the modeling. A simple approach was used for estimation of typical values (tv) and their associated SE and coefficient of variation as an indication of the precision of the estimate. The goodness-of-fit of the model was evaluated by using different diagnostic plots such as visual predictive check, population predicted value based on population parameter estimates (PRED), and individual predictive value based on individual’s etas (IPRED) versus the dependent variable (DV), conditional weighted residuals (CWRES), and the fitting of the individual curves. 

The following formula [31,32] for estimation of T > MIC was applied:

%*f*T > MIC = Ln(C_max_/MIC) × 1/k_10_ × 100/τ (9)

where C_max_ is maximum plasma concentration, MIC is a minimum inhibitory concentration, k_10_ is elimination rate constant, and τ is the dosing interval.

## 5. Conclusions

The developed population pharmacokinetic approach showed relatively low inter-individual variability in the absorption of doxycycline and higher variability in the volume of distribution. The tested co-variates could not improve the model and could not explain between-subject variability. The population pharmacokinetic model can be further developed by addition of data from rabbits. This preliminary study indicates that higher doses have to be tested in future experiments.

## Figures and Tables

**Table 1:** Population primary and secondary parameters of orally administered doxycycline hyclate (5 mg/kg) in rabbits at different ages.

Parameters	Parameter Name	Estimates	Units	SE	CV%	2.5% CI	97.5% CI
Thetas (typical value)	tvka	0.257	1/h	0.033	12.87	0.192	0.323
	tvV	4.429	L/kg	0.702	15.85	3.038	5.821
	tVCl	1.473	L/kg/h	0.080	5.46	1.313	1.632
	stdev0	0.368	µg/mL	0.028	7.61	0.313	0.424
Secondary parameters	k_10_	0.332	1/h	0.049	14.86	0.23	0.43
	Half-life k_10_	2.09	h	0.049	14.86	1.47	2.70
	AUC_0-__∞_	3.40	µg·h/mL	0.185	5.46	3.02	3.76

Typical value (tv) of ka, absorption rate constant; V, volume of distribution; Cl, clearance; stdev, standard deviation for multiplicative residual error; k_10_, elimination rate constant; half-life, k_10_-elimination half-life; and AUC, area under the curve.

**Table 2:** Random effects of orally administered doxycycline (5 mg/kg) in rabbits at different ages.

Omega.	Variance	SE	BSV (CV%)	Shrinkage
ηka	0.103	0.004	32.96	0.300
ηCl	0.033	0.009	18.18	0.246
ηV	0.392	0.129	69.30	0.179

Variance ηka, ηCl, and ηV are random components of the model (eta) and BSV is the between subject variability estimated according to the Equation (6).

## Data Availability

Data is contained within the article and supplementary material.

## Supplementary Materials

The following are available online at https://www.mdpi.com/2079-6382/10/3/310/s1, Table S1: Mean ± SD plasma concentrations of doxycycline in mature (*n* = 6) and immature (*n* = 10) rabbits. Table S2: Individual and mean ± SD values for biochemical parameters of mature and immature rabbits.
