# Supplementary material for: Population Pharmacokinetic Modelling of Orally Administered Doxycycline to Rabbits at Different Ages
Source: Antibiotics (Basel). 2021 Mar 17;10(3):310. doi: 10.3390/antibiotics10030310 (PMC8002702; doi:10.3390/antibiotics10030310)
Supplement: Supplementary file 1 [file antibiotics-10-00310-s001.pdf]

Table S1. Mean ± SD plasma concentrations of doxycycline ($\mu g*mL^{-1}$) in mature (n=6) and immature (n=10) rabbits.

Time (h)	Mature rabbits	Immature rabbits
0.5	0.17±0.11	0.25±0.35
1	0.22±0.09	0.42±0.35
2	0.27±0.12	0.36±0.28
3	0.35±0.13	0.45±0.28
4	0.56±0.20	0.73±0.68
6	0.25±0.08	0.33±0.16
8	0.17±0.08	0.26±0.10
10	0.18±0.08	0.20±0.04
12	<LOQ (0.13±0.05)	0.17±0.11
24	<LOD	< LOD

Blood samples were obtained from 6 immature rabbits at each time interval.

Table S2. Individual and mean ± SD values for biochemical parameters of mature and immature rabbits.

Group of rabbits	Total protein ($g.L^{-1}$)	Albumin ($g.L^{-1}$)	AST (UI)	ALT (UI)	LDH (UI)
		Immature rabbits (70 days old)			
No 1	57.9	27.6	46	77	250
No 2	72.9	28.6	34	69	258
No 3	60.5	26.8	44	81	333
No 4	64.5	29.3	40	87	256
No 5	62.7	27.3	42	63	412
No 6	59.6	26.8	45	77	306
No 7	64.1	29	51	93	257
No 8	61.3	30.2	41	59	236
No 9	67.1	29.3	44	58	436
No 10	61.8	28	49	69	291
No 11	74.9	28.6	64	81	302
No 12	72.9	29.5	37	65	193
Mean ± SD	65.02±5.7	28.42±1.11	44.75±7.69	73.25±11.17	294.17±70.99
		Mature rabbits (5 months old)			
No 1	77.6	35.3	60	34	323
No 2	65.7	30.3	36	52	376
No 3	72.4	33.9	39	26	340
No 4	66.5	28.4	19	20	270
No 5	65.8	29.6	23	41	268
No 6	71.9	30.9	31	32	341
Mean ± SD	69.98±4.81	31.4±2.65*	34.67±14.54*	34.17±11.29*	319.67±42.87

* Statistically significant differences at $p<0.05$, evaluated by Mann-Whitney U test (STATISTICA for Windows 10.0, StatSoft, Inc., USA).
